# Pore-Forming Proteins from Cnidarians and Arachnids as Potential Biotechnological Tools

**DOI:** 10.3390/toxins11060370

**Published:** 2019-06-25

**Authors:** Esperanza Rivera-de-Torre, Juan Palacios-Ortega, José G. Gavilanes, Álvaro Martínez-del-Pozo, Sara García-Linares

**Affiliations:** 1 Departamento de Bioquímica y Biología Molecular, Facultad de CC. Químicas, Universidad Complutense de Madrid, 28040 Madrid, Spain; esperanza.rivera.detorre@gmail.com (E.R.-d.-T.); juan.palaciosb1a@gmail.com (J.P.-O.); jggavila@ucm.es (J.G.G.); 2 Biochemistry, Faculty of Science and Engineering, Åbo Akademi University, 20500 Turku, Finland; 3 Cell Biology Department, Harvard Medical School, Boston, MA 02115, USA

**Keywords:** venomics, transcriptomics, pore-forming proteins, actinoporin, latrotoxin, immunotoxin, bioinsecticides

## Abstract

Animal venoms are complex mixtures of highly specialized toxic molecules. Cnidarians and arachnids produce pore-forming proteins (PFPs) directed against the plasma membrane of their target cells. Among PFPs from cnidarians, actinoporins stand out for their small size and molecular simplicity. While native actinoporins require only sphingomyelin for membrane binding, engineered chimeras containing a recognition antibody-derived domain fused to an actinoporin isoform can nonetheless serve as highly specific immunotoxins. Examples of such constructs targeted against malignant cells have been already reported. However, PFPs from arachnid venoms are less well-studied from a structural and functional point of view. Spiders from the *Latrodectus* genus are professional insect hunters that, as part of their toxic arsenal, produce large PFPs known as latrotoxins. Interestingly, some latrotoxins have been identified as potent and highly-specific insecticides. Given the proteinaceous nature of these toxins, their promising future use as efficient bioinsecticides is discussed throughout this Perspective. Protein engineering and large-scale recombinant production are critical steps for the use of these PFPs as tools to control agriculturally important insect pests. In summary, both families of PFPs, from Cnidaria and Arachnida, appear to be molecules with promising biotechnological applications.

## 1. Introduction

Venomous organisms have fascinated humankind throughout history. This has been, in great part, due to their inherent danger and the fatal consequences exerted by a usually small but envenomed injury. Venomous animals use their venoms for competitive, defensive, and predatory purposes and comprise a phylogenetically diverse set of organisms: from vertebrates like amphibians, reptiles, or even mammals, to invertebrates like insects, arachnids, or cnidarians. Most of these venomous animals are small in size compared to their natural predators and, in many instances, do not seem to be dangerous at first sight.

Venoms are complex mixtures of bioactive compounds such as peptides, proteins, salts, and neurotoxins [1]. Thus, venoms balance the predator–prey relationship. A continuous race is established between both actors: prey are evolutionarily selected for venom resistance, which is achieved through different mechanisms [1,2,3]; to work within the new evolutionary landscape, venoms must adapt quickly to keep harming their prey. How do new venoms appear in a species? One hypothesis is that a proto-toxin gene is first duplicated, and then one copy becomes expressed in the organism’s venom gland. Additional amplification of the venom gland-expressed gene can generate variants that produce a toxic protein product or compound. Due to this duplication–neofunctionalization mechanism, it is common to find venom toxins genetically structured as multigene families [2,4,5,6].

**Figure 1:**
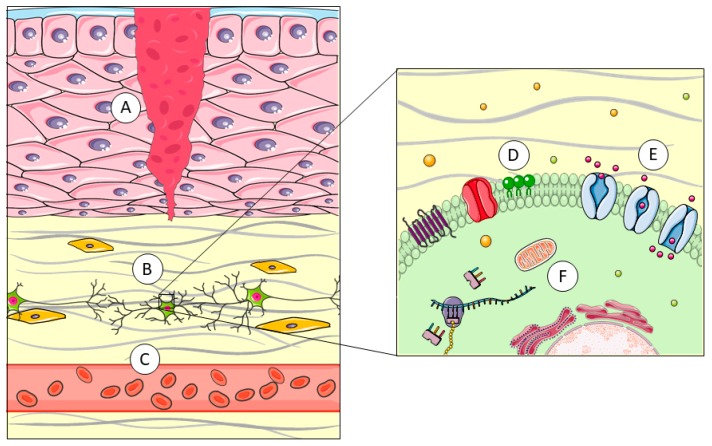
Piercing delivery systems cross the skin, the first barrier encountered by venom (**A**). Specific extra-cellular matrix proteases, like metalloproteases, digest scaffold proteins (**B**). Non-proteinaceous components, like serotonin, promote vasodilatation (**C**). The plasma membrane is the most widespread structure in nature and, therefore, a suitable target of attack by enzymes like phospholipases or cytolysins (**D**). Neurotoxins interact with membrane receptors and channels, leading to imbalance in ion distribution across the membrane (**E**). Some toxins develop their harmful effects once they are internalized, blocking protein production or oxidative respiration in mitochondria (**F**). This image was created using Servier Medical Art free images database (SERVIER, Paris, France).

When venoms are delivered, they must interact with their prey at the molecular level in order to cause cellular damage. Venoms must breach multiple barriers to reach their targets, including skin, the intricate network of the extracellular matrix, and, eventually, the cell membrane. Delivery systems like chelicerae, nematocysts, and other piercing structures have an initial harmful physical activity, overtaking the barriers and bringing toxins closer to their final target [3] (Figure 1). Within the injected cocktail, metalloproteases, for example, are very effective in attacking the interstitial space between cells, by digesting extracellular matrix components and facilitating the activity of other toxins on the cells. Non-proteinaceous components of the venom, like serotonin and histamine, produce fast and sharp pain and increase vasodilatation. As cells are defined by the plasma membrane [7], the first cellular structures encountered by the venomous cocktail are the transmembrane and peripheral receptor proteins as well as the lipid membrane itself. Therefore, venoms also contain proteins that remodel and directly attack the lipid membrane, such as neurotoxins, phospholipases, and pore-forming toxins (PFTs), which finally destroy this key structure defining the cell.

## 2. Pore-Forming Proteins (PFPs)

PFPs are the perfect example of toxins whose activity relies on the disruption of a lipid membrane. In general terms, they violate the standard classification of proteins—into water-soluble or membrane proteins—that can be found in any basic Biochemistry textbook. PFPs remain stably folded and soluble in water but, upon interaction with the membrane of their target cells—and after recognition of a receptor that can be a sugar, a protein, or even a specific lipid—they polymerize into an oligomeric transmembrane protein that makes a pore. We, and other authors, even speak of a molecular metamorphosis transforming these toxins from water-soluble to transmembrane proteins, escaping the aforementioned classification [8,9,10]. Attachment to the membrane increases the local concentration of the toxin, reducing protein diffusion to a bidimensional system, and thus facilitating the oligomerization that leads to pore formation. Depending on the toxin, pore size and permeability selectivity vary, allowing the passage of species ranging in size from small ions to medium-size proteins. In most cases, the final outcome of the formation of a pore is cell death by osmotic shock. Based on the protein structure that forms the final pore, these proteins are classified either as α-PFPs, if the pore walls are defined by α-helices, or β-PFPs, if the pore walls are β-sheets [11,12].

PFPs attack the plasma membrane, the primordial structure that defines a cell. As stated, some of them only need a lipidic receptor, like cholesterol [13] or sphingomyelin [14,15,16,17,18]. But others seem to need protein receptors, such as the understudied latrotoxin macrocomplexes from black widow spiders (*Latrodectus* spp.) [19]. Especially when targeting a particular lipid or lipidic composition, PFPs are not specific enough. However, since they constitute a component of a complex cocktail (the venom), they can be used to target a wide range of animals. These toxins act very quickly [10,12,14], so they are used for both predatory and defensive purposes [1].

In this Perspective, we focus on PFPs produced by two different groups of animals, which constitute our major areas of research in the field. Cnidarians, given their privileged evolutionary position and their wide-range spectrum of attack, and arachnids, whose powerful PFP collection is usually aimed, with high specificity, at insects. Taking into account the above outlined possibilities on toxin biotechnological applications, both groups of proteins display different interesting characteristics to turn them into powerful weapons.

### 2.1. Pore-Forming Proteins in Cnidaria

Cnidaria is an ancient clade of animals, whose genetic analysis is interesting from the evolutionary and phylogenetic point of view because they also are the oldest lineage of venomous animals [2]. Cnidaria includes about 10,000 species, most of them living in saltwater. The Cnidaria phylum is phylogenetically divided in two subdivisions: the class Anthozoa (anemones and corals) and the subphylum Medusozoa, which includes the classes Cubozoa (jellyfishes) and Hydrozoa (hydras), among others. Although they exhibit very simple anatomy, they are able to defend themselves with high rate of success [2]. This animal group has clinical relevance from the point of view of envenomation, and its hazard for humans fluctuates from non-hazardous to extremely dangerous like the Australian box jellyfish (*Chironex fleckeri*) [20] or the Atlantic Portuguese Man o’ War (*Physalia physalis*) [21]. Most sea anemones, however, are harmless to humans or just cause skin burning after contact with tentacles [22].

Sea anemones inject venom into their prey through nematocysts, specialized penetrant structures that discharge upon activation of cnidocytes [1,2]. Nematocysts are mostly located in the tentacles, structures that cover a large area of anemones’ bodies and contain the venomous weapon needed not only to protect the animal, but also to attack and entrap the prey. However, it is also possible to find nematocysts surrounding the oral disc, in order to paralyze the prey, or in the ring surrounding the base of the column, specialized for inter- and intraspecific competition. In fact, Cnidaria is the only venomous lineage that lacks a centralized venom system [23,24].

Like most animal venoms, sea anemones’ venoms can be largely classified as non-proteinaceous toxins, neurotoxins, enzymes, or cytolysins [3,4,6]. Among the latter, actinoporins are a family of α-PFTs produced by sea anemones as part of their venomous arsenal. They are small (around 20 kDa/175 amino acids), cysteineless, and basic (pI ≈ 9) [10,12,25]. It has been largely accepted that actinoporins do not need a protein receptor to exert their toxicity, but instead require sphingomyelin as a specific lipidic receptor [16,17,26,27,28]. Furthermore, cholesterol, though not indispensable, plays a key role in their pore-forming mechanism [14,29,30,31,32,33,34], a mechanism still not fully understood, especially with regard to the sequence of events during pore formation and the final stoichiometry of the pore [35,36,37,38,39,40,41,42]. Overall, actinoporins represent a simple and optimal model to study the challenging biophysical transition from a water-soluble conformation to an integral transmembrane state.

**Figure 2:**
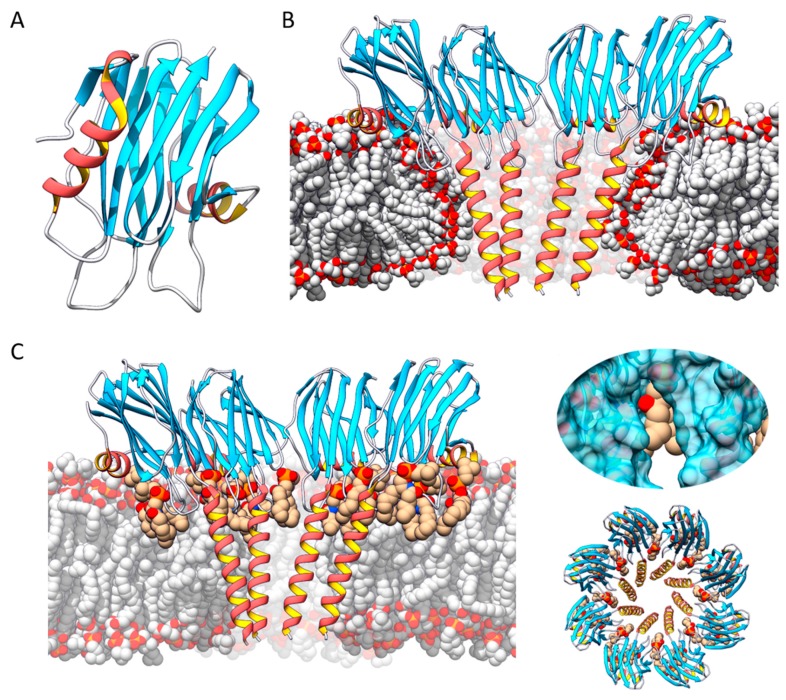
(**A**) Actinoporin monomers share a common fold: a stranded β-sandwich flanked by two short α-helixes. With regard to pore structure, two models have been proposed: (**B**) a tetrameric structure in which the lipid membrane adopts a toroidal shape around the pore walls, and (**C**) an octameric lipid-protein mixed structure in which lipids (in tan color) are accommodated in pore-wall fenestrations (see inserts on the right).

So far, actinoporins have been detected in at least 20 different species of anemones [10,12,25], constituting multigenic families in practically all cases [6,43,44,45]. All of them show a remarkable sequence identity that reaches values as high as 90% [12,25]. But despite this great structural similarity, they also show notable functional differences [14,46]. This identity of sequence is logically manifested in its three-dimensional structure [36,37,42,47,48,49]. All studied actinoporins contain a core consisting of a stranded β-sandwich which is flanked by two short α-helixes. This sandwich contains a motif that anchors the protein to the membrane and remains virtually unchanged during the process of pore formation; in contrast, the N-terminal end, in association with the N-terminal ends of adjacent monomers, is the portion that undergoes a conformational change and finally pierces through the membrane [50] (Figure 2).

The mechanism by which actinoporins carry out their action has been studied in detail and, while some aspects remain controversial, there is at least a general consensus about the stages leading to pore formation [10,12,16,25,51,52]. According to the commonly accepted model, the monomeric units of the protein bind to its target membrane, initiating an oligomerization process followed by separation and elongation of the α-helix located at the N-terminal end of the actinoporin [53,54,55]. It is approximately the first 30 amino acids that adopt a helical amphipathic structure, insert into the membrane, and, in association with other actinoporin molecules, form a pore in the lumen of which the polar heads of some lipids would also participate (Figure 2) [23,36,38,42,50,56,57]. However, there are still aspects of this mechanism to be elucidated: mainly, the order in which these stages occur [8,41,42,51,58], the need for ‘pre-pore’ type intermediates [38,58], and, above all, the stoichiometry and composition of the final functionally active structure [36,37,41,42,51].

At least three different models have been proposed to explain the actinoporins’ transmembrane pore. The first proposed model suggested the existence of a toroidal tetrameric protein-lipidic structure [35,38,59]. However, a non-toroidal nonameric pore of fragaceatoxin C (FraC), produced by the sea anemone *Actinia fragacea*, was proposed based on a detergent-containing crystalline structure [37]. The most detailed structural study carried out in the field is a crystalline lipid-containing octameric pore, solved with atomic resolution [42]. In fact, none of these models is fully accepted and, in our opinion, most probably all of them describe different aspects of the mechanism of pore formation and cell lysis employed by actinoporins. The different conformations detected are just static images of a dynamic process (Figure 2).

### 2.2. Pore-Forming Proteins in Arachnids

Araneae (spiders) is the largest order within the Arachnida class, which also includes scorpions, mites, and harvestmen. Most adult arachnids have eight legs and they also count with other mobile appendages like pedipalps, which are adapted for a wide variety of functions from feeding to reproduction, and chelicerae, involved in feeding and defense. In most venomous arachnid species (*Latrodectus* spp. are just one of the few exceptions), chelicerae are only connected to glands where venom is produced, as opposed to Cnidaria, where it is usually more homogeneously distributed within their bodies. Production of the venom is mediated by a process called holocrine secretion, in which the toxic compounds are produced in the cytosol of the gland cells and, then, these productive cells disintegrate, dumping the cytosol content into the venom gland lumen.

Most of natural spiders’ prey are insects. Consequently, their venoms have been selected during evolution so that they immobilize and kill this particular kind of invertebrate. However, spider venom can be also harmful for vertebrates, a feature most probably ‘developed’ as a protective weapon against predation. Species from the *Latrodectus* genus, commonly known as black widow spiders, constitute a group of around 40,000 different spider species. Their bite causes acute pain and severe secondary conditions in humans, called ‘latrodectism’ [60], which involves a complex symptomatology from nausea to body rigidity and widespread intense pain [61]. Because of the frequency of envenomation events on humans, severity of clinical symptoms, and the frequent serious clinical consequences of their bite, black widow spiders are classified as medically important [62].

#### 2.2.1. Latrotoxins

From the point of view of this Perspective, the most interesting group of toxic proteins from the venom of species belonging to the *Latrodectus* genus are latrotoxins. They are high molecular weight (110–140 kDa) and acidic proteins whose toxic activity relies on the formation of pores through biological membranes [63,64,65,66]. The latrotoxin family includes 3 different subclasses based on their prey specificity: vertebrates (LTX), crustaceans (LTC), or insects (LIT). LTX and LCT subclasses contain only one member each, α-latrotoxin (α-LTX) and α-latrocrustaceatoxin (α-LCT), respectively. So far, five latrotoxins have been identified as insect specific and, consequently, they are known as α, β, γ, δ and ε-latroinsectotoxins (LITs) [67,68]. Therefore, the main difference between α-LTX and the different LITs is prey selectivity. From the evolutional point of view, the presence of different LIT isoforms is related to hunt specialization. Nevertheless, only α-LTX is specific for mammals, which can be associated with the evolution of a defensive weapon against other attackers, like rodents.

α-LTX is the best characterized member of the latrotoxin family and neurexins and latrophilins have been identified as its potential protein receptors [19,63,64]. Although no specific information about LIT receptors has been reported so far, there are orthologs of the above receptors in insects, suggesting that these similar insect proteins could be involved in recognition. However, both α-LTX and LITs have been proven to produce pores not only in cells expressing these receptors, but also in artificial bilayers [19,69]. Interestingly, although very little is known about the physiological activity of δ-LIT, it seems to be receptor-independent, showing a lack of neuronal selectivity.

**Figure 3:**
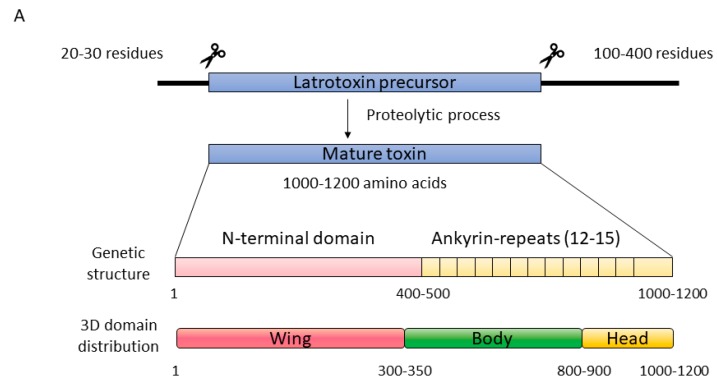

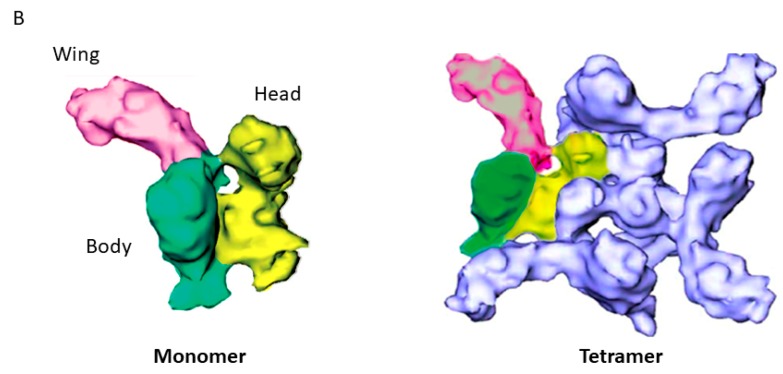
(**A**) Latrotoxins are produced as inactive precursor activated upon proteolytic digestion in both the N- and C-termini. The genetic structure comprises a unique N-terminal domain and a C-terminal domain rich in ankyrin repeats. Within a low-resolution three-dimensional structure of α-LTX obtained by cryo-EM, three different domains can be differentiated: the wing (pink), the body (green), and the head (yellow). (**B**) The final pore is composed by four α-LTX monomers, combined in a so called ‘four-bladed propeller’. The head and the body are responsible for membrane binding and pore formation, while the wing seems to be implicated in receptor recognition. Reproduced from Ushkaryov, Y.A., Volynski, K.E., Ashton, A.C., The multiple actions of black widow spider toxins and their selective use in neurosecretion studies. *Toxicon* 2004, Elsevier.

LTXs are synthetized as large inactive polypeptide precursors which undergo post-translational processing on both their N- and C-termini [63,67,69,70]. The mature versions show a modular structure with three well-differentiated domains: the ‘wing’, the ‘body’, and the ‘head’ [19] (Figure 3). The wing corresponds to a unique N-terminal domain which seems to be involved in receptor recognition, while the C-terminal domain is composed by a high number of consecutive ankyrin repeats and comprises the body and the head. A low-resolution three-dimensional structure was obtained for α-LTX by cryo-electron microscopy (cryo-EM). It appeared as a dimer in the absence of cations but, in the presence of Ca^2+^ or Mg^2+^, ankyrin-repeats probably mediate protein-protein interactions that led to the formation of water-soluble homotetramers with a well-defined central channel. This type of assembly is known as the ‘four-bladed propeller’ model [19] (Figure 3). In fact, this four-bladed structure, which is amphipathic, appears to be inserted into the membrane, forming pores that could also be detected by cryo-EM [19,63]. The reconstruction published had very low resolution [19] but, even so, the presence of ‘windows’ (fenestrations) was observed in the pore lumen. The polar heads of some phospholipids would presumably show up through those fenestrations, providing stability and selectivity to the channel. This situation is far from being solved, but it has also been detected in other PFPs such as the actinoporin FraC [42] (Figure 2). These cryo-EM experiments were carried out, however, not only in the absence of the corresponding membrane protein receptors, but also using palmitoyl-oleyl-phosphatidylcholine (POPC) as the sole support. Plasma membranes also contain large amounts of other key lipids for the physiological behavior of a biological membrane, such as cholesterol (50%), phosphatidylserine (7%), sphingomyelin (4%), and phosphatidylinositols (1%). To date, the role of lipids in the mechanism of action of LTXs is still not fully known. Thus, although the authors of the paper concluded that the toxin itself was capable of forming pores in the membrane in the absence of any other protein component of neuronal origin it is far from proven that another component of spider venom, for example, is not needed to carry out this insertion. The structure-function relationship of these proteins is still mostly unknown at the molecular level. Furthermore, there is no three-dimensional structural information about LITs. However, α-LTX and LIT share the same genetic domain structure; they are around 50% identical, so it can be assumed that they probably exhibit a similar fold.

The physiological effect observed regarding the toxicity of LTXs or LITs in insect tissues includes the increase in the frequency of glutamatergic and GABA-ergic potential at the neuromuscular junctions and the asynchronous release of these neurotransmitters [67,69]. The cholinergic sensory nervous system of the insects is also affected. All these events seem to be related with the major molecular event of LTX toxicity: the formation of cation selective pores in membranes. Thus, in the presynaptic space, pore formation leads to Ca^2+^ fluctuations, promoting massive neurotransmitter release. However, it is still necessary to study LITs in deeper detail in order to understand their toxicity and structure-function relationships. Actually, all the aforementioned LITs have been tested for toxicity against *Galleria mellonella* larvae [71], a model system where they showed very different LD_50_ values (mg of toxin necessaries to kill 50% of the tested animals expressed per kg of body weight).

#### 2.2.2. Latrodectins

Low molecular weight proteins (originally called LMW, or black widow low molecular weight venom components) of around 70 amino acids long and with a high content of disulfide bridges have also been detected within the venomous black widow spider cocktail, and even isolated in small quantities. Nowadays, these proteins are known as latrodectins (Ltds) [72]. Their natural function is not yet known, but the few available results suggest that they are essential for increasing the neurotoxic capacity of LTXs, most likely by cooperating with them and increasing their affinity for the membrane. Paradoxically, their participation seems to be also key in diminishing the specificity of this interaction, thus facilitating an insecticidal activity of α-LTX [72], a toxicity which, in the absence of Ltds, seems to be only restricted to vertebrates. The association between LTXs and Ltds is presumed to be crucial, given that it seems practically impossible to purify LTXs to homogeneity by conventional methods [73,74,75], as they seem to be always contaminated with Ltds. Some authors even consider them as mere subunits of what is called the *latrotoxin macromolecular complex* [74], given that, separately, they do not appear to be toxic neither against insects or vertebrates [75,76,77,78]. As for their structure, apart from their sequences, there is only one spectroscopic characterization, by means of circular dichroism, which suggests that they should have a high α-helix content [79]. Thus, although they are not proven to be PFPs, they could be determinant for the final pore formation of LTXs, forming a macromolecular complex, and their presence should be taken into account when designing biotechnological application of LTXs.

## 3. Biotechnological Applications of PFPs

The obvious benefit of studying venoms in detail is finding the means to block their deleterious effects. Although envenomation is a neglected public health problem in many of developed countries [80], it frequently causes intense pain and, when complicated symptoms occur, can even lead to death of the affected individual. This Perspective, however, focuses on the potential of some venom components as biotechnological tools.

The study of venoms can then open the gate to not-so-obvious venues. For example, detailed knowledge about the mechanism of action of many toxins offers the possibility of using them for at least three different approaches: detecting, inactivating, or modulating different cellular or metabolic pathways. As mentioned above, toxins co-evolve with their targets. During this intense competition, toxic compounds become highly specific to their target. Such a great specificity can be used to fight different pests with the employment of the corresponding venomous natural product. In addition, venom can interfere with the immune system regulating signaling pathways, tuning cytokine secretion or promoting cell migration. Understanding how they work can put toxins on the radar of new immunotherapies with venom or venom-derived products [61,62,81,82].

Given the high specificity and binding affinity of some toxins, they can be also used as molecular probes, after the appropriate modifications such as conjugation to fluorophores. In fact, one of the most successful and best systems for cellular in situ detection of sphingomyelin and cholesterol-rich domains is based on the employment of modified versions of actinoporins, PFTs from sea anemones [83,84].

On the other hand, if the target cellular components or metabolic pathways are dysregulated during a disease, having a molecule with high binding affinity is a perfect starting point to make the appropriate modifications and modulate the target activity, regulating its biological function. Our present knowledge allows molecular mimicry of natural products which appear promising in order to find new therapeutic treatments.

Another possibility would be providing proteinaceous toxins with the means to change their specificity without losing their lethal properties, as in the case of immunotoxins (IMTXs). This approach takes advantage of toxins’ highly specific and potentially deadly activity, but it drives it toward aberrant cells like cancer, metastatic, or cancer stem cells, for example. IMTXs are chimeric constructions built from a target domain, which recognizes the harmful cell, and a toxic domain, which kills it [81,85,86,87,88,89,90,91].

### 3.1. The Biotechnological Potential of Cnidaria PFTs as IMTXs

**Figure 4:**
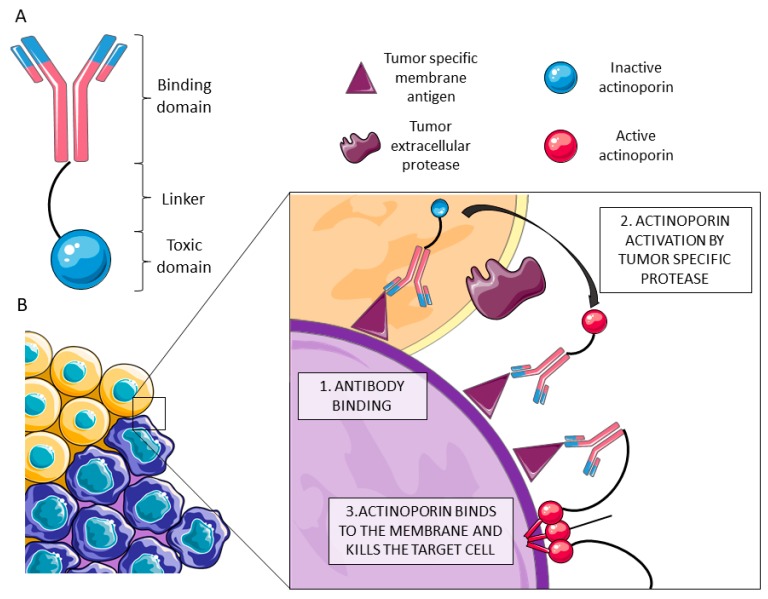
(**A**) Immunotoxins (IMTXs) are chimeric molecules composed of a monoclonal antibody that recognizes the malign cells and a toxin moiety that kills the targeted cells. (**B**) In order to improve the specificity of actinoporin IMTXs, protease-activated variants have been designed. Once the chimeric molecule binds specifically to malign cells (in purple) by recognition of a membrane motif missing in the healthy ones (in orange) (**1**), the toxic moiety is activated by a tumor specific protease (**2**). The activated actinoporin is then able to bind the membrane, oligomerize with other monomers, and form a pore, killing the targeted cell by an osmotic shock (**3**). This image was created using Servier Medical Art free images database (SERVIER, Paris, France).

Immunotoxins are hybrid artificial molecules in which the killer action of a toxin is directed to a target cell through a binding domain (Figure 4). The binding domain can be a monoclonal antibody or, in order to improve the penetration capacity of the construct, a smaller engineered version such as the single-chain variable fraction of the selected antibody. Regarding the toxic moiety, most IMTXs use toxic proteins which, acting intracellularly, lead to cell death by different means. The translocation of this IMTX is then necessary to achieve the internalization of this toxin moiety into the cytosol. This approach is especially successful against hematological tumors [92]. However, IMTXs have trouble attacking solid tumors due to difficult penetration in tumor masses.

One of the advantages of using PFPs as the toxic moiety of IMTXs be is that they do not need internalization to exert their toxic activity. Some examples can be found in recent scientific literature, which use actinoporins [39], melittin peptide from bee venom [93], or the N-terminal domain of the human perforin [94]. Since PFPs increase membrane permeabilization, in addition to their lethal membrane altering properties, they can also facilitate the action of regular chemotherapies by facilitating the entrance to the cytosol [95].

The first IMTX built with an actinoporin was developed by Avila et al. [96] conjugating a monoclonal antibody recognizing IOR-T6, a specific antigen expressed on the surface of immature T-lymphocytes, and a hemolytic toxin from *Stichodacthyla helianthus*. Later, the same group developed a new chimera with a monoclonal antibody against carcinoembryonic antigen (CEA) [97]. One of the latest approximations was linking, again, an actinoporin from *S. helianthus* with a monoclonal antibody recognizing a colorectal cancer-associated antigen (IOR-C2) [98]. Although the chimeras recognized the corresponding antigens preferentially through the monoclonal antibody fraction, the non-specific toxicity against cell lines not expressing the targeted antigen was still relatively high. Thus, the greater advantage of these toxins (attacking the membrane, which is the most widespread target in the living cells), is also their greatest weakness. Further research is required in order to eliminate the off-target effects of the actinoporin-based chimeras.

In addition, once bound, actinoporins need to diffuse within the membrane in order to oligomerize and form a pore. Being attached to an antibody recognizing a surface antigen is probably an obstacle for the final pore formation. However, actinoporins are very well studied from the structure-function point of view, allowing protein engineering to overcome these hurdles. Several research studies delve into the details of the protein-lipid interactions [10,15,30,31,33,42,99,100,101,102,103], making possible the application of different strategies directed to improve toxin specificity: for example, protecting the toxin region responsible for membrane binding or blocking the N-terminal domain directly implicated in pore formation. Actinoporins can be engineered to protect their key regions with polypeptide domains, which can later be released by tumor specific proteases. Actinoporins are quite resistant to protease activity and are cysteineless. This last feature makes them suitable for site directed mutagenesis followed by modification through conjugation. Matrix metalloproteases (MMPs) are extracellular proteases that remodel the extracellular matrix and whose expression is increased in the cells surrounding tumors [104], along with other proteinases like catepsin B [105] or furin [106] (Figure 4). High expression of extracellular matrix proteases is correlated with the invasion capacity of the tumor. Protective domains could be linked to the protein by sites suitable for tumor-specific protease digestion. Conjugating the specificity of the antigen recognition provided by the monoclonal antibody moiety and the proteinase-activated toxic activity could improve the specificity of cytolysin-based IMTXs [95,107,108].

### 3.2. The Biotechnological Potential of Arachnid PFPs as Bioinsecticides

Although the structural and the associated functional knowledge about LITs is restricted and further work has to be done to understand the activity of these toxins, the toxicity information detailed above and the structural characterization of α-LTX make LITs a suitable starting point to design bioinsecticides.

The world population has been increasing exponentially since 1900. By 2050, it is estimated to reach 9 billion, according to the United Nations. Obviously, this increase comes together with a greater need for sustenance. However, crop losses due to plagues are a major problem, worsened every day by climate change [109]. For decades, approximately since the 1940s, pest control relied on the use of dichlorodiphenyltrichloroethane (DDT). It was cheap, easy to produce and deliver, and effective, but its poor selectivity and high toxicity forced many countries to ban it. Within this context, bioinsecticides have emerged as a promising pest control tool [81], based on the use of natural toxins. Spiders, scorpions, and many other venomous animals have insects as preferred prey. Therefore, their toxic cocktails include compounds specifically directed to insects, which appear highly valuable to fight pests [110,111]. Thus, these biopesticide approaches would include two different strategies: the development of suitable tools for fumigation or the construction of transgenic variants which constitutively produce protective toxins to avoid damage caused by pests. Although there is still skepticism about the consumption of genetically modified organisms, no adverse effects have been observed so far [112], and proper legislation about their commercialization should be introduced in the near future to take advantage of their proven beneficial properties [113].

The ideal bioinsecticide should be stable in conditions of extreme humidity and temperature, active topically or orally, and should be rapidly lethal to pest insects, but innocuous to humans and other non-pest species like bees, fishes, or birds. In addition, it should not be bioaccumulated or produce harmful secondary or degradation products [111]. A priori, insecticidal toxins from spiders cover almost every one of these requirements, even regarding degradation, since proteins are completely biodegradable into innocuous amino acids.

The most successful example of bioinsecticide is *Bacillus thuringiensis* insecticidal three-domain Cry toxin (3d-Cry) [114], which is in fact a PFP. When a susceptible larva ingests the toxin, it gets activated in the gut, where it binds and leads to pore formation, provoking cell death and compromising larvae viability. The major issues encountered by this approximation is the UV sensitivity of the toxin that reduces its availability for spray dispersion and the emergence of insect resistance [115,116]. As introduced at the beginning of this article, venom effectivity/resistance is a constant race for survival and without insecticidal innovation pest would end up developing resistance.

The toxicity of LTXs, as well as many other components of the spider venom, is exerted at the level of neuromuscular junction. For spiders, reaching the target cells is easier through piercing chelicerae, but applying the toxins alone as biopesticides requires identification of an easier administration/delivery. There are two possible administration routes that imply different technological approaches: intoxication of the insect through ingestion or reaching the neuromuscular junctions through the spiracles connecting to the tracheas that transport the gases and connect with tissues for cellular respiration. Some spider toxins (like disulfide-rich knottin peptides, known as inhibitor-cystine knot (ICK)), have been already demonstrated to be potent, stable and orally active for insects [111,117,118,119]. If LITs were orally active, it would be possible to deliver them in aerosol, which would be the easiest way to commercialize them. However, big proteins are more prone to degradation, and the resistance to extreme humidity or temperature conditions is not guaranteed. Designing transgenic crops constitutively producing the compound is another option that has been successful for other toxins like the aforementioned *B. thuringiensis* toxins [120], which have been proven to be safe for human consumption. In addition, the toxins produced by the transgenic species only affect insects consuming the crop, avoiding the collateral damage to beneficial insects like bees and other critical species for the pollination maintenance. Since LTXs are PFPs and they increase the permeability of the target cells, their application in combination with other bioinsecticide components whose activity is developed intracellularly [81,111] can increase the bioinsecticide activity synergistically.

The greatest inconvenience for insecticide application of LTX family raised by different authors is the high molecular mass of this class of toxins, and therefore, the difficulty of cloning and producing them in heterologous systems [111,121]. However, successful experience cloning and expressing the mature form of δ-LIT in bacteria has been already accomplished [69]. Moreover, according to the preliminary data obtained through the structural information for α-LTX pore, the whole protein is not necessary to promote pore formation. Given the high identity between α-LTX and LITs and assuming that the three-dimensional structure would be similar to that of α-LTX, it might be possible to eliminate the ‘wing’ domain, which is supposed to be implicated in receptor recognition. Although more studies need to be done on the selectivity of LITs, as well as the structural domain implicated in receptor recognition, restricting the cloned sequence to the minimum necessary to exert lytic activity would reduce the specificity of the attack, but it might increase the chances of success. Other approaches to increase the oral activity of toxins is conjugating them with carrier proteins recognized by insect midgut transporters like the mannose-specific lectin agglutinin from *Galanthus nivalis*. Upon ingestion by insects, it is recognized by a receptor located in the midgut cells. It is receptor-mediated endocytosed, followed by transportation and accumulation in the hemolymph [122].

**Figure 5:**
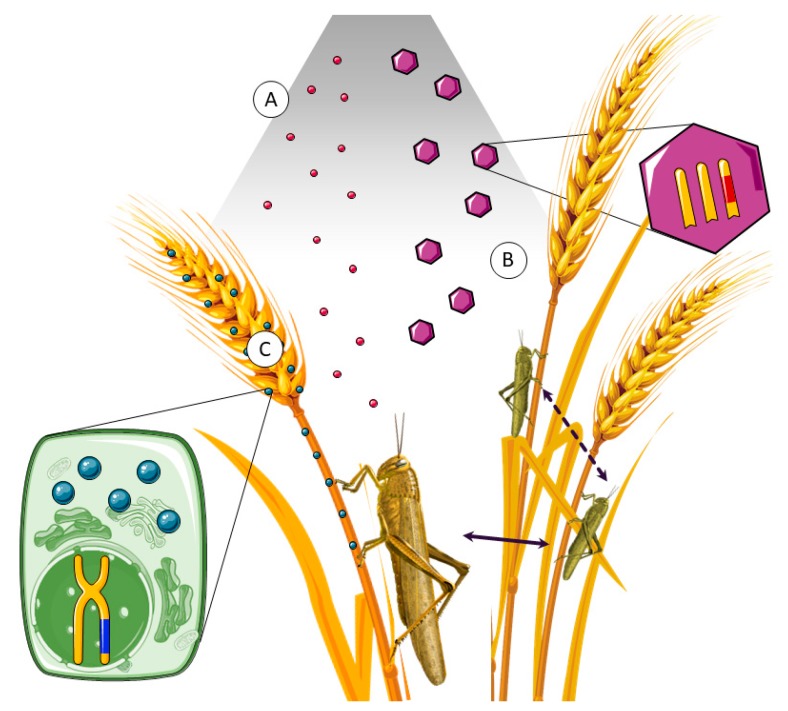
Bioinsecticides can be spread over crops as aerosols if they are orally active, or they can enter their target through insect spiracles. Bioinsecticides can be delivered directly as active toxic products (**A**) or as genetically modified baculovirus-based pesticides, containing toxin genes (**B**). After larvae ingest baculoviruses by feeding, they can develop a lethal disease, releasing new infective particles, suitable for horizontal (dashed arrow) or vertical (continuous arrow) infection. Genetically modified crops for biopesticide production eliminate the pest in both adult and larva state if they are orally active (**C**). This image was created using Servier Medical Art free images database (SERVIER, Paris, France).

Another interesting option is the use of baculoviruses as pest control agents. Natural baculoviruses have been already used for effective pest control. However, genetic engineering amplifies the possibilities of this tool, making them an even better alternative [123,124,125,126]. Natural baculoviruses kill pest by themselves within weeks, while recombinant baculoviruses including toxins would shorten the treatment time. In addition, this system has the advantage of being stable by itself, while also providing specificity for the host and the possibility of treatment maintenance due to the potential transmission of the virus both horizontally (among insects of the same species in the same development state) and vertically (from adult insects to their progeny) [125] (Figure 5). Interestingly, it has been very recently reported how a transgenic fungus expressing a potent insect-selective spider toxin can be effective against insecticidal resistant mosquitos [127].

## 4. *Omic* Techniques in the Discovery of New Potentially Useful PFPs

One of the most famous examples of toxin-inspired pharmacological drugs is captopril, a drug for hypertension treatment that inhibits angiotensin-converting enzyme (ACE). Its discovery was derived from studies with small toxic peptides from the snake *Bothrops jararaca* [128] and it was approved by the FDA in 1981. Research in this field is now booming as a consequence of the technological development of powerful techniques for identification of new bioactive compounds. Identification of new toxins is associated with a great variety of potential applications.

‘Omic’ techniques are instrumental and informatis-dependent tools that have reached new levels of performance within the past decade. As stated at the beginning of this article, venomous animals are usually small and venom yield production is low. These facts were major obstacles in venom research. The purification of bioactive compounds rendered small yields, such that only the more abundant compounds were detected and used in research, even if low-abundance molecules had a critical role in toxicity. Many of these new ‘omic’ approaches have reduced the amount of starting material needed for the analysis, making it affordable for venom studies [129,130,131,132]. In depth analysis of venoms from long-neglected organisms using these techniques not only increases the knowledge about venom composition, phylogeny, and evolution, but also allows the identification of underrepresented toxins which may exert unique biological properties and hold potential applications. These techniques are excellent for high throughput screening of new bioactive compounds. Venoms are a quite fascinating material from the evolutionary point of view, and genomic, transcriptomic, and proteomic analysis reveal quite interesting features about the evolution of venomous species [132,133].

Among all these massive analysis techniques, transcriptomics has arisen as the preferred analysis for this venom specific approximation, providing the most secure approach to assess general venom composition. The strength of genomics is its ability to work with a great amount of data, but de novo analysis is still slow. On the other hand, RNAseq analysis has proven to be a solid approximation for de novo assembly, thanks to algorithms like Trinity [134]. In addition, it provides information about transcript quantification, increasing the value of the obtained information when multi-tissue studies are developed. Dissecting the whole venom delivery system makes a good picture of the full toxin arsenal produced [132,135,136,137]. For a long time, transcriptomics has been the only way to analyze venom-related samples because it was not possible to obtain enough material to apply other high-throughput techniques, such as proteomics, which needs a greater starting material. Indeed, it is still the preferred pre-screening technique [6,135,138]. However, the sample requirements have been reduced by the progress of mass spectrometry (MS) instruments which now provide fragmentation techniques reducing the cost of massive peptide sequencing on tandem MS (MS/MS) [139]. Besides sequence and expression levels, proteomics reveals valuable information about multiple protein products translated from alternatively spliced transcripts and post-translational modifications (PTMs) such as phosphorylation, glycosylation, elimination of signal peptides, or cysteine scaffold patterns [136,139,140]. Indeed, glycomics is a proteomics subdivision which is gaining importance in translational venom research because the identification of glycosylated peptides and proteins can be very important in the identification of compounds suitable for therapeutic application, in particular identification of carbohydrate- and protein-based epitopes as allergic response promoters [136]. Most of the protein sequences annotated as toxins were predicted from RNA sequences with open reading frame-finding tools, they are not supported by any experimental data. Proteomics provides the necessary evidence to verify the transcripts obtained on transcriptomic analysis. Combination of proteomics and transcriptomics is critical in the identification of false-positive putative toxin transcripts [140,141,142].

‘Omic’ techniques come together with an intense bioinformatic progress. Assembly of the huge de novo transcriptomic and genomic jigsaws would not be possible without the appropriate programs [134,143] and the necessary computational power [6,135,143,144]. The availability of in silico applications that predict both the presence of a toxin with a completely new activity or the possible off-target effects that would limit the potential use of the toxin as a biotechnological tool opens up new opportunities for novel drug discovery [145]. In full agreement with this development, the term ‘venomics’ has been established to define the application of ‘omic’ techniques to venoms. Indeed, some authors have developed a standardized workflow for venomics [132,136,146].

Finally, the term ‘pharmacomics’ refers to the holistic focus on ‘omic’, merged with the functional characterization of newly discovered bioactive compounds, extending the classical venomic definition with the critical step of functional characterization [136]. The perspectives in the application of toxins as tools in pharmacology and bioinsecticides are promising, and the emerging techniques combined with bioinformatic advances will facilitate the discovery of new toxins suitable for its use in the biotechnology field.

## 5. Conclusions

Animal venoms have been recognized as a source for bioactive compounds such as peptides and proteins with potential use in different biotechnological fields. PFPs are a common family of proteins present in the vast majority of venoms. They share a widespread target, the plasma membrane, and promote cell death through osmotic shock and depolarization of the plasma membrane target cells. Their lytic properties can be resourceful if directed to specific targets, as well as in increasing their permeability, easing the access to other toxic components acting at the intracellular level. Actinoporins produced by sea anemones do not need a protein receptor, but SM, a lipidic receptor. This feature makes them wide-spectrum toxins. The biotechnological applications for this kind of PFPs include the design of probes for SM and SM-rich domains, and protein engineering for construction of immunotoxins against cancer cells or parasites. Regarding immunotoxins, although promising strategies have been applied to improve selectivity of the toxic moiety for the targeted cells, further work has to be done. On the other hand, spiders are professional insect hunters whose venomous arsenal is a potential source of insecticide compounds. Among their toxin arsenal, latrotoxins are a family of big PFPs that promote ion depolarization and massive neurotransmitter release at the neuromuscular junction of insects, provoking their paralysis and death within seconds. This kind of toxin is attractive for the development of new bioinsecticides that lack the already shown resistance of other biopesticides like the one based on the 3d-Crys toxins produced by *B. thuringiensis*. However, LITs suffer the lack of structural and functional characterization as well as the evidence of oral activity for their use as sprays or as part of transgenic crops, therefore extensive and detailed research has to be done to harvest beneficial applications.

The emergence and establishment of accessible high-throughput screening technology makes easier to find new bioactive compounds with unknown activities that can be used in new applications, always taking into account the native activity of the toxin. Transcriptomics is the most widely used technique for the search of new compounds, although the establishment of more complex workflows for venom identification and functional annotation is improving the accuracy and increasing the range of possibilities.
